# How growth mindset influences mathematics achievements: A study of Chinese middle school students

**DOI:** 10.3389/fpsyg.2023.1148754

**Published:** 2023-03-28

**Authors:** Lianchun Dong, Xiaoying Jia, Yaxin Fei

**Affiliations:** ^1^College of Science, Minzu University of China, Beijing, China; ^2^Shenzhen Zhenneng School, Shenzhen, Guangdong, China

**Keywords:** growth mindset, fixed mindset, mathematics, achievements, attributions, intrinsic motivation, mathematics self-efficacy, mathematics anxiety

## Abstract

**Introduction:**

It has been suggested that students with growth mindsets are more likely to achieve better mathematics learning results than their counterparts with fixed mindsets. However, inconsistent and some even contradictory results have been reported in recent studies which examined the associations between growth mindset and mathematics achievements, suggesting the complexity regarding the effects of growth mindset on academic achievements.

**Methods:**

This study aims to examine students' growth mindsets, failure attributions, intrinsic motivation, mathematics self-efficacy, mathematics anxiety and mathematics achievements in one model to capture the sophisticated functioning processes of growth mindset. A total number of 266 middle school students in China participated in this study. Students' mindset and related variables (i.e.,motivations to learn mathematics, attributions of failure in mathematics, mathematics anxiety, mathematics self-efficacy) were measured at year 7, the first year of junior middle school in China. These students' mathematics learning outcomes were tracked from year 7 to year 9, the end of junior middle school. Structural equation modeling (SEM) was used to investigate the relations among students' growth mindsets, failure attributions, intrinsic motivation, mathematics self-efficacy, mathematics anxiety and mathematics achievements.

**Results:**

The results show that: (1) growth mindset doesn't directly predict mathematics achievements; (2) growth mindset indirectly influences mathematics achievements through intrinsic motivation; (3) failure attributions and mathematics self-efficacy sequentially mediate the association between growth mindset mathematics achievements; (4) failure attributions and mathematics anxiety sequentially mediate the relationship between growth mindset mathematics achievements.

**Discussion:**

The results of this study contribute a better understanding about how growth mindsets make impacts on middle school students' mathematics achievements. These findings have important implications for mathematics education in that we could not simply cultivate students' growth mindset in schools with expectations of higher mathematics learning outcomes. Instead, along with the growth mindset intervention, it is fundamental to make interventions on students' intrinsic motivation, failure attribution, mathematics self-efficacy, and mathematics anxiety in mathematics teaching and learning.

## 1. Introduction

The theory of growth mindset has attracted researcher's interest in the past decade. A growth mindset refers to the belief that one person's ability can be developed through efforts, whereas a fixed mindset means viewing ability as fixed and unchangeable (Yeager and Dweck, 2012; Xu et al., 2022). During the last decades, there has been a controversy regarding whether a growth mindset could predict mathematics learning outcomes (Burnette et al., 2013; Yeager and Dweck, 2020). Some researchers reported a positive association between a growth mindset and mathematics achievement (Blackwell et al., 2007), whereas other studies showed no or negative correlations between these two variables (Bahník and Vranka, 2017; Li and Bates, 2019; Yeager and Dweck, 2020).

These results prompted researchers to reconsider the growth mindsets' predictive role in students' mathematics learning. To obtain further insights into the above discrepancies, researchers proposed to conduct further investigations to understand the sophisticated mechanisms in which growth mindsets and related variables (e.g., motivation and attributions) function together to influence academic outcomes (Burgoyne et al., 2020; Yeager and Dweck, 2020). Thus, it is necessary to examine multiple related variables and their mutual connections together in one study. This study aims to investigate the effects of a growth mindset on students' mathematics achievement by considering failure attributions, intrinsic motivation, mathematics self-efficacy, and mathematics anxiety together.

## 2. Literature review

This section presents previous studies involving the connections between a growth mindset and mathematics achievements and the impacts of failure attributions, intrinsic motivation, mathematics self-efficacy, and mathematics anxiety.

### 2.1. Failure attribution in mathematics learning and growth mindset

Failure attributions refer to students' inclination to attribute their academic failure or setbacks to possible impacting factors. Failure attributions orient students toward different patterns of responding to failure and setbacks in learning (e.g., whether students take remedy strategies or give up) and thereby have significant impacts on academic outcomes (Weiner, 2010; Organisation for Economic Co-operation and Development and Programme for International Student Assessment, 2019).

It is reported that students with growth mindsets tend to interpret academic failure and setbacks differently from those with fixed mindsets (De Castella and Byrne, 2015; Martin et al., 2017). When reflecting on the cause of failure, students with growth mindsets usually focus on controllable characteristics (e.g., insufficient efforts). For example, Hong et al. (1999) reported that students with growth mindsets are more likely to make effort attribution, believing the cause of failure is their lack of effort. By contrast, students with fixed mindsets tend to attribute failure to uncontrollable aspects (e.g., the lack of ability). Previous studies reported that fixed mindsets are significantly associated with ability attribution in the failure contexts, indicating that students with fixed mindsets perceived low ability as the reason for failure (Robins and Pals, 2002; Tempelaar et al., 2014; Smiley et al., 2016). The ability attribution is also called helpless attribution by Yeager and Dweck (2020), who claimed that students with a fixed mindset are inclined to attribute their undesirable performances to a stable flaw and thereby show helpless behavior when facing academic setbacks.

### 2.2. Mathematics self-efficacy and growth mindset

Self-efficacy refers to individuals' beliefs about their abilities to achieve a certain level of performance in specific activities or tasks (Bandura, 1977). Self-efficacy has been reported to make an impact on students' mathematics learning processes and outcomes (Hackett and Betz, 1989; Pajares and Kranzler, 1995; Zimmerman, 2000; Huang et al., 2019; Organisation for Economic Co-operation and Development and Programme for International Student Assessment, 2019). Researchers pointed out that growth and fixed mindset are significantly correlated with students' mathematics self-efficacy (Young and Urdan, 1993; Abdullah, 2008; Todor, 2014; van Aalderen-Smeets et al., 2018). It was reported that higher levels of mathematics self-efficacy are more likely to be observed when students adopt a growth mindset, whereas students with fixed mindsets usually have lower levels of mathematics self-efficacy (Todor, 2014). van Aalderen-Smeets et al. (2018) claimed that the adoption of a growth mindset could help students to maintain a relatively stable level of self-efficacy when facing failure and difficulties in learning (van Aalderen-Smeets et al., 2018). In contrast, students with a fixed mindset tend to believe failure is the result of a lack of capability rather than effort, which usually leads to a decline in self-efficacy.

Davis et al. reported that a fixed mindset could result in the feeling of helplessness when facing setbacks and challenges, which in turn decreases mathematics self-efficacy (Davis et al., 2010). Recent intervention studies reported that growth mindset intervention could improve students' self-efficacy (Samuel and Warner, 2019; Zhang et al., 2022). For example, a two-semester intervention based on a growth mindset was executed in a college statistics course. At the end of the intervention, students in the intervention group reported a higher level of mathematics self-efficacy than those in the control group (Samuel and Warner, 2019).

The roles of self-efficacy are also underlined when investigating the processes of how growth and fixed mindset impact on academic learning (Burgoyne et al., 2020). Some researchers conducted a regression analysis to confirm that a growth mindset positively predicts self-efficacy and a fixed mindset negatively predicts self-efficacy (BrÅten et al., 2005). Some researchers claimed that students with fixed mindsets responded differently to failure, depending on the level of students' self-efficacy (Dweck and Leggett, 1988; Gonida et al., 2006). In general, low persistence and challenge avoidance are more likely to be observed when students are more inclined to believe intelligence is unchallengeable. However, if these students have a higher level of self-efficacy, they are less likely to hesitate when facing an academic challenge. Instead, these students with a higher level of self-efficacy, despite their tendency toward a fixed mindset, could embrace challenges in academic learning and maintain persistence when experiencing failure.

Researchers also reported that self-efficacy plays a mediating role between a growth mindset and academic achievements. Leondari and Gialamas (2002) found that a growth mindset promotes students' orientation toward learning goals, leading to a higher level of self-efficacy, which in turn enhances academic achievements (Leondari and Gialamas, 2002). Other studies reported that a growth mindset leads to higher academic achievements through the chain mediating effects of mathematics self-efficacy and beliefs of failure (Su et al., 2021).

Failure attributional style is closely linked with self-efficacy (Schunk, 1981; Wang et al., 2008; Siegle et al., 2009; Cheng and Chiou, 2010). Students' self-efficacy declines as a result of attributing failure to stable and uncontrollable factors (e.g., the ability of students with fixed mindsets; Silver et al., 1995). By contrast, when making adaptive attribution for academic failure (e.g., attributing failure to the lack of effort), students can develop their self-efficacy and believe their academic performance can be improved by continuous efforts (Baird et al., 2009).

Further studies show that there is a chain-mediating role of effort beliefs, failure attribution, and positive remedy strategies in the connections between a growth mindset and academic achievements (Blackwell et al., 2007). However, Cheng and Chiou (2010) reported that the level of self-efficacy can be weakened when students attribute failure to personal factors, regardless of whether it is personal ability or personal efforts. By contrast, students' self-efficacy can be improved when attributing failure to situational factors, such as the difficulty level of a test and luck.

### 2.3. Mathematics anxiety and growth mindset

Mathematics anxiety refers to students' feelings of anxiety and tension when interfering with mathematics knowledge and tasks (Richardson and Suinn, 1972; Essau et al., 2008; Radišić et al., 2014; Dirzyte et al., 2021; Li et al., 2021). Mathematics anxiety usually leads to the avoidance of mathematics tasks and activities (Hembree, 1990; Dowker et al., 2016), disrupts students' attention and working memory (Cohen and Rubinsten, 2017), and results in poor mathematics performances (Wang et al., 2015; Byrnes and Miller-Cotto, 2016; Ramirez et al., 2018; Kaskens et al., 2020; Geary et al., 2021).

Mathematics anxiety is also related to failure attributions. Researchers reported that students attributing failure in mathematics to ability tend to have a high level of mathematics anxiety (Arkin et al., 1983; Hunsley, 1987). Dweck and Licht (1980) also suggested that failure attributions can explain the observed gender differences regarding learning anxiety. Further studies investigated the effects of failure attributions on mathematics anxiety, presenting that effort attributions predicted a low level of mathematics anxiety in exam contexts, whereas ability attributions led to a high level of mathematics anxiety (Bandalos et al., 1995).

In addition, researchers also claimed that students' anxious experiences in mathematics learning are always related to their perceptions of personal abilities (Clark, 2021; Young and Dyess, 2021). Intervention studies also reported that developing students' growth mindset in mathematics classrooms can successfully relieve mathematics anxiety and increase mathematics achievements (Boaler, 2013; Smith and Capuzzi, 2019; Clark, 2021; Young and Dyess, 2021). These findings support the assumption that a growth mindset might influence mathematics achievements through attributions and mathematics anxiety.

### 2.4. Intrinsic motivation to learn mathematics and growth mindset

Learning motivation refers to the psychological processes that drive students to engage in learning activities and specific tasks. In general, there are two types of the motivation behind mathematics learning: learning mathematics because of individual enjoyment and interest (i.e., intrinsic motivation) and learning mathematics due to external rewards (i.e., extrinsic or instrumental motivation; Ryan and Deci, 2009; Wigfield et al., 2009; Organisation for Economic Co-operation and Development and Programme for International Student Assessment, 2019). Compared with extrinsic motivation, intrinsic motivation is more likely to contribute to high-quality learning behavior (e.g., high-level engagement, more persistence, and efforts) and outcomes (e.g., deep understanding and high academic achievements; Murayama et al., 2012; Taylor et al., 2014; Gottfried, 2019; Karlen et al., 2019).

Previous studies show that students' mindsets can make an impact on their learning motivation (Molden and Dweck, 2000; Dweck, 2002). Students with growth mindsets tend to have a stronger intrinsic motivation to learn, whereas learners with fixed mindsets are more likely to be motivated by external rewards (Blackwell et al., 2007; Komarraju and Nadler, 2013; Tempelaar et al., 2014). Blackwell et al. (2007) conducted an intervention to promote students' growth mindset, reporting that students in the experimental group showed larger improvements in intrinsic motivation than those in the control group. This finding was supported by other intervention studies, which highlighted that effective mindset interventions contributed to maintaining a high level of students' motivation to learn mathematics (Priess-Groben and Hyde, 2016).

Researchers also reported that the link between a growth mindset and better academic results could be explained by considering the effects of a growth mindset on learning motivations. Compared with those with a fixed mindset, students with a growth mindset usually focus more on skill improvements by learning, and thereby are inclined to maintain a high level of intrinsic motivation, which results in better learning behavior and outcomes, especially when facing challenging learning tasks (Burnette et al., 2013; Karlen et al., 2019). Degol et al. (2017) found that students holding a malleable perspective of mathematics intelligence tend to have more appreciation for the value of mathematics and for personal connections with mathematics and develop more intrinsic motivation to engage in mathematics learning, which ultimately leads to higher mathematics achievements.

## 3. The present study and research hypotheses

**Figure 1:**
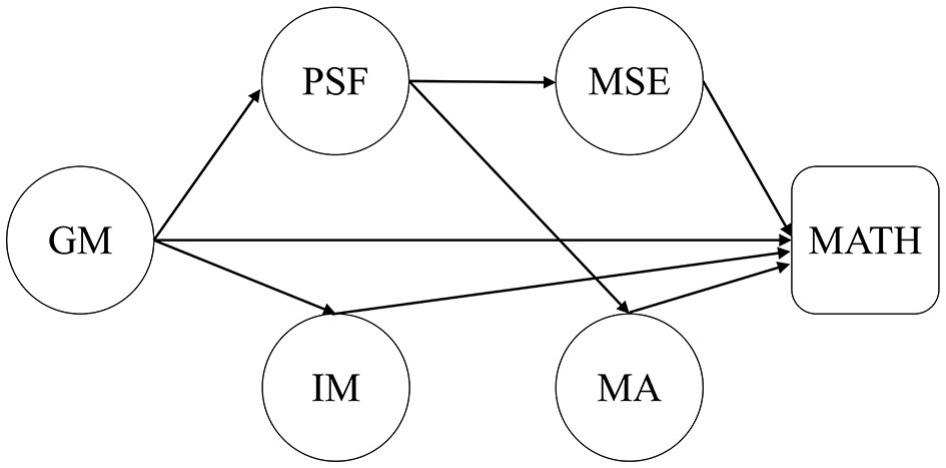
Conceptual research model. GM, growth mindset; MSE, mathematics self-efficacy; IM, intrinsic motivation to learn mathematics; MA, mathematics anxiety; PSF, perceived self-responsibility for failing in mathematics; MATH, mathematics achievement.

This study aims to include growth mindset, failure attribution, intrinsic motivation, mathematics self-efficacy, and mathematics anxiety together to examine the processes in which a growth mindset influences mathematics achievements. Based on the above discussion, a conceptual research model is established to demonstrate the hypothesized relationships (see Figure 1). The research hypotheses in this study are outlined below.

H1: A growth mindset would predict mathematics achievements. Students with growth mindsets would have higher mathematics achievements than students with fixed mindsets.

H2: Intrinsic motivation to learn mathematics would mediate the relationship between a growth mindset and mathematics achievement. Students with growth mindsets are more likely to have stronger learning motivation than their peers with fixed mindsets and, thereby, have better learning results in mathematics.

H3: There would be a significant sequential mediation path from growth mindset to perceived self-responsibility for failing in mathematics, to mathematics self-efficacy, and then to mathematics achievement. Compared with students with fixed mindsets, students with growth mindsets are more inclined to attribute undesirable mathematics learning results to themselves (e.g., efforts) rather than external factors (e.g., luck). Therefore, students with growth mindsets have a better feeling of control over their mathematics learning, which leads to higher mathematics self-efficacy, believing that they would improve their mathematics learning by making more effort. This could, in turn, contribute to better mathematics learning results.

H4: There would be a significant sequential mediation path from a growth mindset to perceived self-responsibility for failing in mathematics, to mathematics anxiety, and then to mathematics achievement. As stated in H3, a growth mindset leads to the tendency to attribute undesirable mathematics learning results to oneself (e.g., efforts) rather than external factors (e.g., luck). This attribution style can reduce students' mathematics anxiety because the efforts to improve learning mathematics can be controlled by students. With a lower level of mathematics anxiety, students are more likely to make higher mathematics achievements.

## 4. Materials and methods

### 4.1. Participants

This study selected junior middle school students from one middle school in Hengshui City, Hebei Province, China. We advertised our research projects to a couple of middle schools in Hebei Province. But because this study requires the tracking of students' achievements over the period through years 7–9, most of the middle schools could not make sure whether they could complete the collections of students' achievements in such a long period of time. Therefore, only one middle school agreed to participate in this study and was interested in examining the effects of students' growth mindset on mathematics achievements over students' whole middle school life.

This junior middle school is located in the urban region of the city, consisting of 3-year levels, respectively years 7, 8, and 9. In China education system, students have 6 years of primary education, followed by 3 years of junior middle school study, i.e., years 7, 8, and 9. At the end of year 9, students sit for a high-stake test that determines whether students can be admitted by senior middle schools.

At the end of year 7, participants filled in the scale of growth mindset, mathematics self-efficacy, motivation to learn mathematics, mathematics anxiety, and perceived self-responsibility for failing in mathematics. Then we tracked participants' mathematics learning achievements from years 8 to 9. Participants' mathematics achievements at three points were collected: the end of year 8, the end of the first semester of year 9, and the end of the second semester of year 9. We did not collect data about students' ages, but according to the Chinese education system, school education from years 1 to 9 is compulsory for all children, and thereby all participants in the study were 12 or 13 years old in year 7.

A total number of 266 students participated in all rounds of data collection. Only students participating in all rounds of data collection were included in the data analysis.

### 4.2. Instruments

#### 4.2.1. Mathematics achievements

When exampling the effects of a growth mindset on mathematics achievements, one of the issues raised by researchers is the limitations of the cross-sectional design adopted in most of the previous studies. The measurement of student mathematics achievements at one single point in time might constrain the observation of the long-term impacts that growth mindsets might exert on students' mathematics learning. Thus, this study attempted to collect students' achievement data at multiple points.

Participants' mathematics achievements were measured by mathematics assessments designed and administered by the local education department. The items of mathematics assessments involved the mathematics facts, skills, and problem-solving strategies required in the National Mathematics Curriculum Standards issued by the Ministry of Education in China. Three mathematics tests' results were collected separately at the end of year 8, the middle, and the end of year 9. Each test lasted 2 h and had a total score of 120. An average of the three tests' results was used to measure students' overall mathematics achievements in junior middle school study.

#### 4.2.2. Growth mindset

The growth mindset measure was adopted from Dweck et al.'s (1995) and Dweck (1999) work. Five items are included in the growth mindset scale. Two items are growth mindset statements (e.g., “You can always greatly change how intelligent you are”) and the other three are fixed mindset statements (e.g., “Your intelligence is something about you that you can't change very much”). The 6-point Likert responses were used, and participants needed to select one choice from 1 (strongly disagree) to 6 (strongly agree). The fixed mindsets items were scored reversely. Scores on the six items were added altogether as an overall growth mindset score, with higher scores indicating stronger beliefs in a growth mindset. The reliability of this measure was α = 0.79 (*N* = 266).

#### 4.2.3. Mathematics self-efficacy

Mathematics self-efficacy measure was adopted from the context questionnaire in PISA 2009 survey (Organisation for Economic Co-operation and Development, 2010; PISA 2009 Shanghai Committee, 2016). This measure included seven mathematics tasks with different levels of cognitive load (e.g., “Solving an equation like 3*x* + 5 = 17,” “Using a train schedule to figure out how long it would take to get from one place to another”). Participants were asked to express their confidence in solving these tasks by selecting a choice from 1 (not at all confident) to 4 (very confident). The sum of scores on all seven tasks was used as the overall measure of mathematics self-efficacy, with higher scores representing a higher level of mathematics self-efficacy. The reliability of this measure was α = 0.87 (*N* = 266).

#### 4.2.4. Intrinsic motivation to learn mathematics

Intrinsic motivation to learn mathematics measure was adopted from the context questionnaire in PISA 2009 survey (Organisation for Economic Co-operation and Development, 2010; PISA 2009 Shanghai Committee, 2016). This measure included four items describing the motivation in mathematics learning (e.g., “I do mathematics because I enjoy it”). Participants need to select one choice from 1 (strongly disagree) to 4 (strongly agree). The sum of scores on all four items was used as the overall measure of intrinsic motivation to learn mathematics, with higher scores representing stronger intrinsic motivation. The reliability of this measure was α = 0.89 (*N* = 266).

#### 4.2.5. Mathematics anxiety

Mathematics anxiety measure was adopted from the context questionnaire in PISA 2009 survey (Organisation for Economic Co-operation and Development, 2010; PISA 2009 Shanghai Committee, 2016). This measure included three items about mathematics learning experiences (e.g., “I often worry that it will be difficult for me in mathematics classes”). Participants need to select one choice from 1 (strongly disagree) to 4 (strongly agree). The sum of scores on all three items was used as the overall measure of mathematics anxiety, with higher scores indicating a higher level of anxiety in mathematics learning. The reliability of this measure was α = 0.86 (*N* = 266).

#### 4.2.6. Perceived self-responsibility for failing in mathematics

In order to measure students' failure attributions, perceived self-responsibility for failing in mathematics measure was adopted from the context questionnaire in PISA 2009 survey (Organisation for Economic Co-operation and Development, 2010; PISA 2009 Shanghai Committee, 2016). This measure provides a scenario about participants' undesirable results in mathematics quizzes and asks participants whether they agree with six given statements (e.g., “I'm not very good at solving mathematics problems”). Participants need to select one choice from 1 (very likely) to 4 (not at all likely). The sum of scores on all six items was used as the overall measure of perceived self-responsibility for failing in mathematics, with higher scores indicating a higher tendency to attribute undesirable mathematics learning results to oneself. The reliability of this measure was α = 0.79 (*N* = 266).

### 4.3. Data analysis

SPSS 22.0 and Mplus 7.0 were used for data analysis. Correlation analysis was conducted to examine the relationship between different measures. Structure equation modeling with latent variables was used to test the mediating effects of the motivational variables (i.e., mathematics self-efficacy, intrinsic motivation to learn mathematics, mathematics anxiety, and perceived self-responsibility for failing in mathematics) in the relationship between growth mindset and mathematics achievement.

## 5. Results

### 5.1. Descriptive statistics

Table 1 presents descriptive statistics, and Table 2 shows the results of correlation analyses. It can be seen that all variables are intercorrelated significantly except that between growth mindset and mathematics achievement. Both growth mindset and mathematics achievement are positively correlated with mathematics self-efficacy, intrinsic motivation to learn mathematics, and perceived self-responsibility for failing in mathematics but negatively correlated with mathematics anxiety.

**Table 1:** Summary of descriptive statistics.

**Measures**	***N***	***Min***	***Max***	***Mean***	***SD***
Growth mindset	266	5	30	22.81	5.09
Math self-efficacy	266	7	28	23.86	3.85
Intrinsic motivation to learn math	266	4	16	12.02	2.63
Math anxiety	266	3	12	7.27	2.30
Perceived self-responsibility for failing in math	266	6	24	17.60	3.27
Math achievement	266	9	110	51.57	25.0

**Table 2:** Summary of correlation analyses.

	**1**	**2**	**3**	**4**	**5**
1. Growth mindset					
2. Math self-efficacy	0.21^**^				
3. Intrinsic motivation to learn math	0.36^**^	0.49^**^			
4. Math anxiety	−0.17^**^	−0.42^**^	−0.38^**^		
5. Perceived self-responsibility for failing in math	0.33^**^	0.52^**^	0.69^**^	−0.51^**^	
6. Math achievement	0.10	0.49^**^	0.42^**^	−0.37^**^	0.45^**^

** *p* < 0.01.

### 5.2. SEM analyses

#### 5.2.1. Common method biases

Because self-reported items were used in the measurement, Harman's single-factor test was used to assess common method bias. Exploratory factor analysis (EFA) was conducted on all the items. Unrotated EFA shows that KMO (Kaiser–Meyer–Olkin's measure) is 0.90 (>0.8), and χ^2^ for Bartlett's sphericity test is significant (*p* < 0.001). There are five factors with an eigenvalue higher than one, and the first common factor explained 33.1% of the total variance in the variables. This proportion is less than the threshold of 40%, suggesting that the problem of common method bias is not present in the data (see Table 3).

**Table 3:** Harman's single-factor test.

	**KMO**	**χ^2^**	***P***	**Number of factors**	**Explained variance by the first factor**
All items	0.90	3326.93	< 0.001	25	33.1%
Recommended threshold	>0.8	-	< 0.05	>1	< 40%

#### 5.2.2. Structural equation modeling results

**Figure 2:**
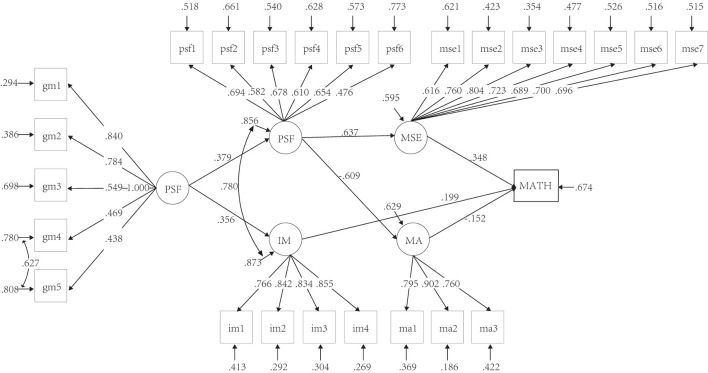
The structural equations model of the relationships growth mindset. GM, growth mindset; MSE, mathematics self-efficacy; IM, intrinsic motivation to learn mathematics; MA, mathematics anxiety; PSF, perceived self-responsibility for failing in mathematics; MATH, mathematics achievement. All paths *p* < 0.05.

Structural equation modeling with latent variables was used to examine the fit of our full model (see Figure 2). Growth mindset was indexed by five items, mathematics self-efficacy was indexed by seven items, intrinsic motivation to learn mathematics was indexed by four items, mathematics anxiety was indexed by three items, and perceived self-responsibility for failing in mathematics was indexed by six items (see the 4.2 section, for detailed explanations about all the items). Mathematics achievement was used as an outcome variable. The full model had an acceptable fit to the data (see Table 4).

**Table 4:** Fit indices of the full model.

	**χ**	***df***	** ${}^{\chi^{2}}{/}_{df}$ **	**RMSEA**	**CFI**	**TLI**	**SRMR**
Full model	510.22	291	1.753	0.053	0.933	0.925	0.058
Suggested threshold	-	-	< 5	< 0.08	>0.9	>0.9	< 0.08

We did not a direct link between a growth mindset and mathematics achievement, which is inconsistent with the hypothesized model (see Figure 1 in Section 3). Actuality, it can be seen in Figure 2 that a growth mindset did not directly predict mathematics achievement, but rather made impacts on mathematics achievement *via* other mediating variables.

A growth mindset positively predicted both perceived self-responsibility for failing in mathematics (β = 0.379, *p* < 0.001) and intrinsic motivation to learn mathematics (β = 0.356, *p* < 0.001). Perceived self-responsibility for failing in mathematics positively predicted mathematics self-efficacy (β = 0.637, *p* < 0.001) and negatively predicted mathematics anxiety (β = −0.637, *p* < 0.001). Mathematics achievement was positively predicted by mathematics self-efficacy (β = 0.348, *p* < 0.001) and intrinsic motivation to learn mathematics (β = 0.199, *p* < 0.05) but negatively predicted by mathematics anxiety (β = −0.152, *p* < 0.05).

#### 5.2.3. Mediation analysis

The full model suggests indirect effects of a growth mindset on mathematics achievements through three mediational pathways: (1) the sequential mediation effect from growth mindset to perceived self-responsibility for failing in mathematics to mathematics self-efficacy to mathematics achievements; (2) the sequential mediation effect from growth mindset to perceived self-responsibility for failing in mathematics to mathematics anxiety to mathematics achievements; (3) intrinsic motivation to learn mathematics mediates the relationship between growth mindset and mathematics achievements.

The mediation effects were evaluated by the bootstrapping method with 1,000 bootstrap data samples, and the results are shown in Table 5. It can be seen that zero is not included in the 95% confidence intervals, suggesting that all the indirect effects are statistically significant.

**Table 5:** Summary of mediation analysis.

**Path**	**Effect size**	**95% confidence interval**
GM → PSF → MSE → MATH	0.084	[0.047, 0.133]
GM → PSF → MA → MATH	0.035	[0.006, 0.082]
GM → IM → MATH	0.071	[0.020, 0.141]
Total	0.190	[0.118, 0.262]

GM, growth mindset; MSE, mathematics self-efficacy; IM, intrinsic motivation to learn mathematics; MA, mathematics anxiety; PSF, perceived self-responsibility for failing in mathematics; MATH, mathematics achievement.

## 6. Discussion

### 6.1. A growth mindset does not directly predict mathematics achievements

This study shows that a growth mindset does not directly predict mathematics achievements, indicating that H1 is not supported. This is not consistent neither with the mindset theory proposed by Yeager and Dweck (2020) nor with the evidence of the direct link between a growth mindset and academic achievements (Costa and Faria, 2018). However, this finding is in line with the results reported by some other researchers (e.g., Li and Bates, 2019; Burgoyne et al., 2020). This study supports the claim that the connections between a growth mindset and mathematics achievements are sophisticated, depending on such factors as cultural contexts (Yeager and Dweck, 2020; Dong and Kang, 2022).

By citing the PISA data (Schleicher, 2019), Yeager and Dweck (2020) claimed that the links between a growth mindset and achievement might be the weakest in Chinese contexts compared with other cultures, such as Western contexts. The weak link was attributed to the Chinese culture of valuing diligence and efforts, and thereby there might be little space to get improvements in study hours or exam scores resulting from the adoption of a growth mindset. In order to further explain the heterogeneity, Yeager and Dweck (2020) suggested conducting cross-cultural studies to compare the effects of a growth mindset in different cultures.

### 6.2. The mediating role of intrinsic motivation

This study finds that a growth mindset indirectly influences mathematics achievements through intrinsic motivation, supporting H2. This is consistent with the previous findings that students with growth mindset motivation are more likely to maintain strong learning motivation and consequently achieve better results than those with fixed mindsets (Burnette et al., 2013; Degol et al., 2017; Karlen et al., 2019). Previous studies selected high school students in Western contexts and showed that students viewing intelligence as malleable are more inclined to engage in challenging mathematics activities and tasks than students with fixed mindsets (Jones et al., 2011; Degol et al., 2017; Karlen et al., 2019). Therefore, students with growth mindsets are more likely to develop an interest and enjoyment in mathematics learning, which leads to fewer chances to withdraw from mathematics tasks and a higher possibility of obtaining desirable mathematics achievements. By collecting data on middle school students in China, this study provides further evidence that the mediating roles of intrinsic motivation can be observed in the stage of middle school learning in Chinese contexts.

### 6.3. The sequential mediation effect of failure attribution and mathematics self-efficacy

This study shows that a growth mindset indirectly influences mathematics achievements through other factors. It presents that perceived self-responsibility for failing in mathematics and mathematics self-efficacy sequentially mediate the association between growth mindset mathematics achievements, supporting H3. This finding highlights the mediating roles of failure attribution between growth mindset and academic achievements, which is consistent with previous studies (Cheng and Chiou, 2010; Yeager and Dweck, 2020).

This study also reports the mediating role of mathematics self-efficacy in the relationship between growth mindset and mathematics achievements, supporting the previous findings that high mathematics self-efficacy is a significant variable when explaining the process in which growth mindset could influence mathematics achievements (Su et al., 2021). It is noteworthy that Su et al. focused on primary school students in their study, highlighting the roles of mathematics self-efficacy in primary mathematics learning. By observing similar results in the middle school students' samples, this study provides further evidence that the roles of mathematics self-efficacy in mindset theory are generalizable across grade levels.

Another contribution of this study is to report the sequential mediation effects of failure attributions and mathematics self-efficacy, highlighting how these two variables might work together to explain the relationship between growth mindset and mathematics achievements. It is claimed that mindset theory constructs a meaning system to explain discrepancies regarding students' responses to challenging situations and failures (Dweck and Yeager, 2019; Yeager and Dweck, 2020). Failure attributions have been reported to be the core of mindset theory in that different mindsets guide students toward different attributions in the face of failure, which in turn exerts great impacts on students' learning behavior and outcomes (Yeager and Dweck, 2020). However, very few studies consider the roles of attribution and self-efficacy together in one model to examine the functioning mechanisms of a growth mindset (Burgoyne et al., 2020). By reporting the sequential mediation effects of failure attributions and mathematics self-efficacy, this study contributes to a better understanding of the processes of how a growth mindset impact on academic achievements.

### 6.4. The sequential mediation effect of failure attribution and mathematics anxiety

This study also presents that perceived self-responsibility for failing in mathematics and mathematics anxiety sequentially mediate the relationship between growth mindset mathematics achievements, supporting H4. This finding suggests the necessity of including mathematics anxiety in order to better understand the processes by which a growth mindset can influence academic achievement.

The inclusion of mathematics anxiety in this study is based on previous findings that mathematics anxiety is observed to decrease during the interventions aiming to foster students' development of a growth mindset (Smith and Capuzzi, 2019; Clark, 2021; Young and Dyess, 2021). However, when examining the processes in which a growth mindset might influence academic achievements, previous studies (e.g., Yeager and Dweck, 2020) rarely include learning anxiety as a variable in the model, making it unclear whether learning anxiety can help to explain the relationship between growth mindset, attributions, and academic achievements. Thus, this study contributes to show evidence that a growth mindset can shape students' failure attributions, and thereby reduce their mathematics anxiety, which consequently results in better mathematics achievements.

## 7. Conclusion and limitations

This study examined Chinese middle school students' growth mindsets, failure attributions, intrinsic motivation, mathematics self-efficacy, mathematics anxiety, and mathematics achievements in one model, aiming to better understand how growth mindset impact on students' mathematics achievements over the period from years 7 to 9. The findings show that a growth mindset does not directly predict mathematics achievements in middle school study, but rather indirectly influences mathematics achievements through other variables, i.e., intrinsic motivation, failure attribution, mathematics self-efficacy, and mathematics anxiety. These findings have important implications for mathematics education in that we could not simply cultivate students' growth mindset in schools with expectations of higher mathematics learning outcomes. Instead, along with the growth mindset intervention, it is fundamental to make interventions on students' intrinsic motivation, failure attribution, mathematics self-efficacy, and mathematics anxiety in mathematics teaching and learning.

This study has some limitations that need to be considered when interpreting the results. First, the sample included only middle school students in China, and it is unclear whether the same results can be generalized to other cultural contexts. Thus, more investigations in different settings are necessary to test the generalizability of the findings in this study. Second, this study did not consider all the impacting factors related to a growth mindset, such as goal orientations and effort beliefs. Future research is needed to construct a more sophisticated model to consider these factors altogether to investigate their interrelationships. Third, although students' mathematics achievements were measured several times from years 7 to 9, we measured other variables (e.g., mathematics anxiety) only in year 7. Because other variables (e.g., mathematics anxiety) might change over the period, this study could not take into account the changes in these variables and the corresponding impacts on students' mathematics achievements. Further studies can measure these variables several times to track the possible changes in these variables to better understand how mathematics achievements might be influenced by these variables through middle school study.

## Data availability statement

The raw data supporting the conclusions of this article will be made available by the authors, without undue reservation.

## Ethics statement

The studies involving human participants were reviewed and approved by Ethics Committee in College of Science, Minzu University of China. Written informed consent to participate in this study was provided by the participants' legal guardian/next of kin.

## Author contributions

LD designed the study, collected the data, and revised the draft of the manuscript. XJ and YF analyzed the data and drafted the manuscript. All authors contributed to the article and approved the submitted version.
